# Pandemic Influenza (H1N1) 2009 Is Associated with Severe Disease in India

**DOI:** 10.1371/journal.pone.0010540

**Published:** 2010-05-07

**Authors:** Akhilesh C. Mishra, Mandeep S. Chadha, Manohar L. Choudhary, Varsha A. Potdar

**Affiliations:** National Institute of Virology, Pune, India; University of Georgia, United States of America

## Abstract

**Background:**

Pandemic influenza A (H1N1) 2009 has posed a serious public health challenge world-wide. In absence of reliable information on severity of the disease, the nations are unable to decide on the appropriate response against this disease.

**Methods:**

Based on the results of laboratory investigations, attendance in outpatient department, hospital admissions and mortality from the cases of influenza like illness from 1 August to 31 October 2009 in Pune urban agglomeration, risk of hospitalization and case fatality ratio were assessed to determine the severity of pandemic H1N1 and seasonal influenza-A infections.

**Results:**

Prevalence of pandemic H1N1 as well as seasonal-A cases were high in Pune urban agglomeration during the study period. The cases positive for pandemic H1N1 virus had significantly higher risk of hospitalization than those positive for seasonal influenza-A viruses (OR: 1.7). Of 93 influenza related deaths, 57 and 8 deaths from Pune (urban) and 27 and 1 death from Pune (rural) were from pandemic H1N1 positive and seasonal-A positive cases respectively. The case fatality ratio 0.86% for pandemic H1N1 was significantly higher than that of seasonal-A (0.13%) and it was in category 3 of the pandemic severity index of CDC, USA. The data on the cumulative fatality of rural and urban Pune revealed that with time the epidemic is spreading to rural areas.

**Conclusions:**

The severity of the H1N1 influenza pandemic is less than that reported for ‘Spanish flu 1918’ but higher than other pandemics of the 20^th^ century. Thus, pandemic influenza should be considered as serious health threat and unprecedented global response seems justified.

## Introduction

Emergence of the influenza pandemic (H1N1) 2009 (pandemic H1N1) posed new challenges to the public health systems and communities all over the world. Global actions by international agencies and highly vigilant media generated tremendous fear resulting into unprecedented response to the new pandemic by majority of the nations. However, it is not clear that the fear generated was in proportion to the risk that this virus presents.

As of 6 December 2009, worldwide more than 208 countries had reported laboratory confirmed cases of pandemic H1N1, including at least 9596 deaths [1]. It is well established that the virus is highly transmissible as it has spread to large number of countries within a short period of time. However, there are divergent views regarding severity of this disease [2]–[6]. Mexico, Thailand and Japan reported higher severity whereas Canada, USA, Australia and many European countries treat it at par with seasonal influenza. Research on previous pandemic strains of influenza suggested that mortality can vary widely between different countries, with mortality being concentrated in developing countries [7]. It is also argued that pandemic H1N1 should be taken seriously as a public health threat and underestimation of the menace by the virus may be by far more dangerous than the overestimation [8]. Therefore, there is need to conduct systematic epidemiological studies in different real time situations.

The pandemic has created tremendous hardship on already overburdened health system in developing nations but unprecedented surveillance has also provided opportunity to study different epidemiological parameters including disease severity of influenza viruses in tropical settings like India. Understanding of severity is central to health care planning for management of the pandemic in the future and to avoid wastage of resources in resource poor settings. Better estimates will also facilitate identification of risk factors between and within populations and regions. Policy regarding social distancing, school closures, diagnosis of patients, vaccination etc would be greatly influenced by these estimates.

Severity of infection can be assessed by different approaches. One is to explore different genetic markers of the virus that are known to be associated with severe influenza. The current circulating pandemic H1N1 does not contain the known molecular markers of pathogenicity and transmissibility [4], [9]. However, absence of known markers does not necessarily indicate that the virus is benign and previously unrecognized molecular determinants could be responsible for transmission among humans. Another is the epidemiological approach. Several epidemiological methods have been proposed by different workers for estimation of case fatality ratio (CFR) [7], [10], [11]. Recently, Garske et al [12] provided a simple 2 stage method for computation of CFR. The first stage involved a short period, usually in the beginning after declaration of the epidemic, for which reliable data is available on all the cases. This data was used for determination of ratio between the hospitalized cases and the total cases, termed as hospitalization ratio. As epidemic grows, significant under reporting of mild cases may occur as people who have mild infection will not present to health care and this is likely to grow as epidemic advances and pressure on public health increases. Similarly, the attending clinicians, with advancement of epidemic, may lose interest in detection and reporting of all the cases. However, it is believed that the hospitalization and fatality reporting will not be affected in the same manner and proper reporting will continue throughout the season. Thus, the hospitalization ratio will hold good for entire season of the transmission. In the second stage, only hospitalized cases and deaths were used to estimate the case fatality ratio among hospitalized cases. The overall case fatality ratio was estimated by multiplication of the hospitalization ratio calculated in the first stage and the case fatality ratio among hospitalized cases estimated in the second stage.

In India, after declaration of pandemic (phase 6) by World Health Organization (WHO) on 11 June [13], an active surveillance was started for detection of influenza cases in persons with travel history to influenza positive countries. All suspected cases of pandemic H1N1 were detained and hospitalized. Only confirmed cases were provided with antiviral treatment. In Pune, the first pandemic H1N1 positive case was detected on 22 June 2009 in a traveler coming from USA. This was followed by 1 more case in June and 8 cases up to 14 July. All these cases were either persons with foreign travel history or the contacts of such persons. The first death due to pandemic H1N1 was reported on 3 August 2009. Thereafter, the active surveillance in community was started by screening all suspected cases of influenza.

In this communication we have followed the epidemiological approach suggested by Garske et al (2009) to assess severity of infection in pandemic H1N1 and seasonal influenza-A patients.

## Results

### Confirmed cases

Since the beginning of this pandemic, 11 May to 31 October 2009, a total of 607,117 persons presented themselves for screening to various health centers in Pune. Of these 34,917 persons were determined as suspected cases of ILI. From 1 August to 31 October, a total of 7,866 suspected cases were sampled for diagnosis of influenza etiology. Frequencies of pandemic H1N1 and seasonal-A cases were 17.8% and 16.3% respectively (Table 1). Due to heavy work load and priority for identification of pandemic H1N1, all the influenza-A positive cases were not sub-typed. A sub-set of 374 seasonal-A positive samples, selected randomly for testing, yielded 86 (23%) seasonal H1N1, and 288 (77%) H3N2 virus. In a subset of 399 cases, selected randomly, only 6 were positive for influenza-B virus.

**Table 1:** Results of the samples tested in the laboratory from 1 August to 31 October 2009.

Cases	Total Influenza-A			IPD cases			OPD cases		
	Suspect cases	Pandemic H1N1 (%)	Seasonal-A (%)	Suspect cases	Pandemic H1N1 (%)	Seasonal-A (%)	Suspect Cases	Pandemic H1N1 (%)	Seasonal-A (%)
Total	7866	1404 (17.8)	1286 (16.3)	3300	569 (17.2)	424 (12.8)	2967	541 (18.2)	687 (23.2)
Range	8–327	1–97	0–79	7–73	0–21	0–20	0–298	0–75	0–65
Daily median/Average	85.5	15.3	14	35.9	6.2	4.61	32.3	5.9	7.5
95% Confidence interval		17.0, 18.7	15.5, 17.2		16.0, 18.6	11.7, 14.0		16.9, 19.7	21.7, 24.7

### Out-patient Department (OPD) cases

**Figure 1:**
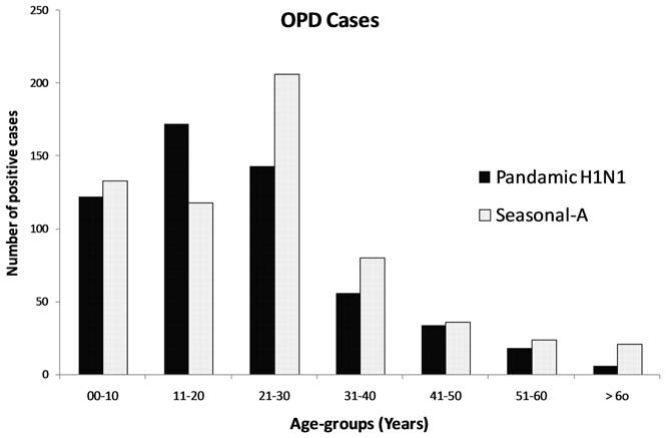
Age distribution of the OPD cases in Pune (Urban).

After the first death on 3 August, regular local screening of all cases of suspected ILI was started. From the second week of September onward, only limited samples, collected at the discretion of the clinicians, were referred to the laboratory because suspected cases were administered Oseltamivir without laboratory confirmation. From 1 August to 31 October 2009, clinical samples from 2967 suspected cases tested in laboratory yielded 18.2% and 23.2% pandemic and seasonal-A influenza respectively (Table 1). Pandemic H1N1 cases were significantly less prevalent than Seasonal-A cases. Among positives, male: female ratio was 1∶1.6. Seasonal-A cases were significantly higher than pandemic H1N1 cases in all age groups except in 11 to 20 years group (Figure 1). A distinct peak in number of positive cases was observed in middle of August but the pattern was not clear afterwards as only limited samples were tested.

### Hospitalized or In-patient Department (IPD) cases

**Figure 2:**
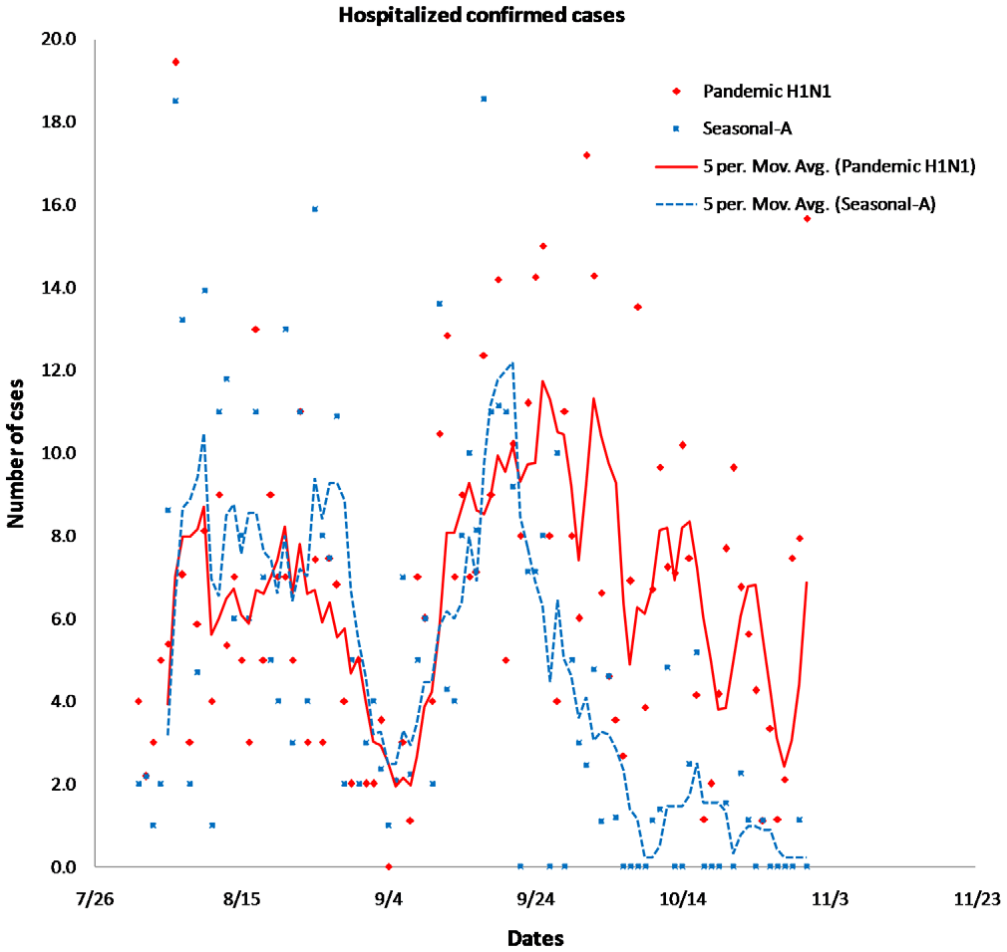
Epidemic curve of the confirmed IPD cases.

**Figure 3:**
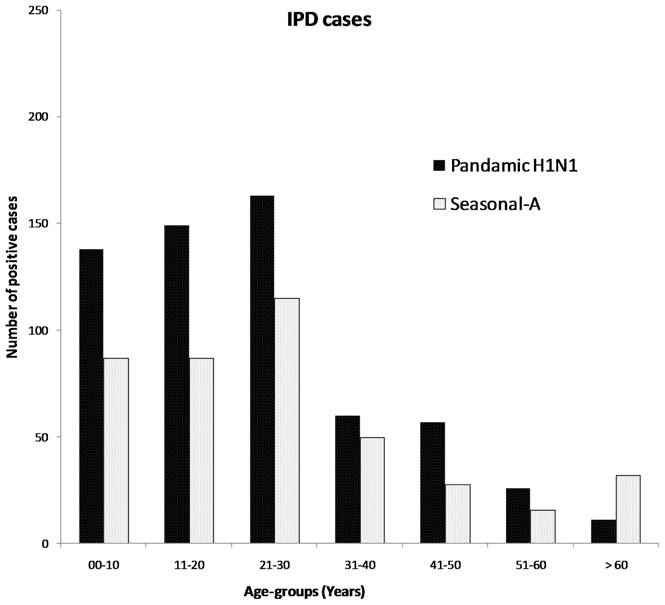
Age distribution of the IPD cases in Pune (Urban).

Between 1 August to 31 October, a total of 3,300 suspected cases were hospitalized (Table 1). Of these, 569 (17.2%) were positive for pandemic H1N1 and 424 (12.8%) for seasonal influenza-A viruses. The epidemic curve for hospitalized cases of pandemic H1N1 and seasonal-A cases is presented in figure 2. Sharp rise was observed in the number of positive cases between 5 and 28 August. Thereafter, influenza activity was very low for about a week from 29 August to 3 September. The second rise in number of cases was observed between 7 and 29 September. In case of pandemic H1N1, the second rise was more stable and comparatively high throughout the remaining part of September and October. The seasonal-A cases declined sharply during the last week of September. Further, number of pandemic H1N1 cases were higher than seasonal-A cases in all age groups except in the group over 60 years (Figure 3). Male: female ratio was 1∶1.4.

### Risk of hospitalization and hospitalization ratio

In IPD, from 1 August to 31 Ocober 2009, out of 993 positive cases, pandemic H1N1 cases (569, 57.3%) were significantly higher than seasonal-A cases (424, 42.7%). However, in OPD, out of 1228 positive cases, pandemic H1N1 cases (541, 44.06%) were significantly less than seasonal-A cases (687, 55.94%) (Table 1). Thus, the odds of hospitalization in pandemic H1N1 positive cases were 1.7 times higher than seasonal-A positive cases [OR 1.7]. In the early stage of the active surveillance, from 4 August to 3 September 2009, the hospitalization ratio was calculated by dividing number of IPD cases by the number of total cases. The ratio for pandemic and seasonal-A influenza was 8.6% and 6.8% respectively. The ratio between hospitalized and OPD cases was 1∶10.7 for pandemic H1N1 and 1∶13.6 for seasonal influenza (Table 2).

**Table 2:** Calculation of hospitalization ratio from 4 August to 3 September 2009.

	Influenza-A	Pandemic H1N1	Seasonal-A
OPD cases	4870	1944	2926
IPD cases	397	182	215
Total cases	5267	2126	3141
Hospitalization ratio IPD/Total cases (%)	7.5	8.6	6.8

### Case fatality

A total of 93 deaths were recorded in Pune district from 1 August to 31 October 2009. Of these, 57 and 8 deaths from Pune (urban) and 27 and 1 from Pune (rural) were among pandemic H1N1 positive and seasonal-A positive cases respectively. Of the 9 deaths associated with the seasonal influenza, 2 were positive for seasonal H1N1 and 7 for H3N2 viruses.

**Figure 4:**
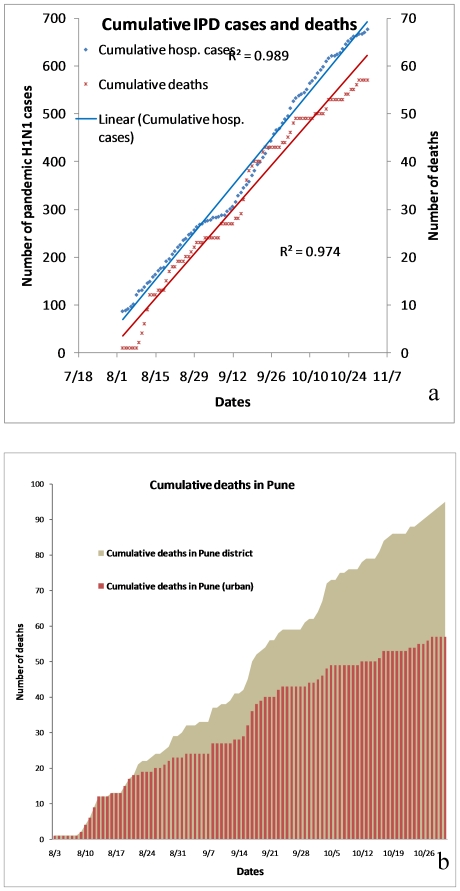
Trends in the cumulative numbers. (a) IPD cases and the deaths in Pune (Urban) (b) Death cases in Pune.

**Figure 5:**
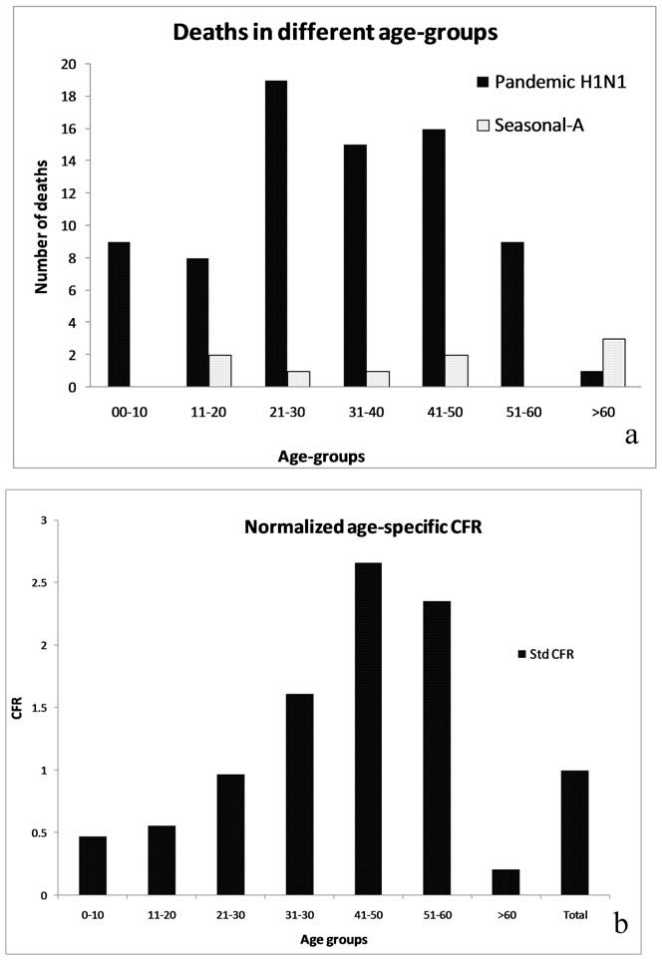
(a) Age-wise distribution of death cases in Pune (b) The normalized age specific CFR.

The number of cumulative deaths in pandemic H1N1 positive cases, presented in figure 4a, show significant linear trend (R^2^ = 0.99). The trend in hospitalization and the trend in fatality were proportionate over a period of time, suggesting about 1 death per 11 confirmed hospitalized cases. Significant linear trends were also observed in cumulative deaths in pandemic H1N1 positive cases in Pune (urban) and Pune (rural) (Figure 4b.). This revealed that with time proportionately more deaths were recorded from rural areas implying higher transmission and spread of pandemic H1N1 in rural areas. This may partly be attributed to the health seeking behavior of the rural population and also issues related to health access. In pandemic H1N1 positive cases, deaths were reported in all age groups, but 75% of all the deaths were in age group of <30 years (Figure 5a).

### Case Fatality Ratio (CFR)

In Pune (urban), the overall CFR for pandemic H1N1 cases (0.86%) was significantly higher than that of seasonal-A cases (0.13%) [P<0.001, OR 5.8, 95% CI (2.71, 13.34)]. The odds of death in hospitalized pandemic H1N1cases was about 6 times higher than seasonal-A cases. As suggested by Garske et al (2009), the precision of the CFR estimates depends upon the sample size of the cases in the first stage of the study. To obtain 95% confidence interval, around 1100 total cases and 200 hospitalizations are required to estimate CFR in the range of 0.5 to 1.5%. Therefore, in this study, the sample size for influenza-A (397 hospitalization and 5267 total cases) and pandemic H1N1 (182 hospitalizations and 2126 total cases) meets the criteria and the estimated CFR of 0.49 and 0.86% respectively can be considered precise at 95% confidence interval. At least 1300 total cases and 500 hospitalizations are needed for estimation of CFR of about 0.2%. The precision at 0.2% CFR cannot be obtained with 200 hospitalizations, no matter how large is the initial sample. Thus, the estimated CFR for seasonal influenza though useful for comparison with the pandemic H1N1, may not represent the true estimate. The CFR for different months of the study period varied between 0.49 and 0.43 for influenza-A, 0.67 and 1.07 for pandemic H1N1 and 0 and 0.19 for seasonal-A (Table 3). The high CFR for pandemic influenza in all the 3 months was a noteworthy feature of the outbreak. Normalized CFR for each age group (Figure 5b) clearly showed a marked increase in fatality risk with age and highest risk was noted in 40–50 years age group.

**Table 3:** Calculation of case fatality ratios.

	Influenza-A	Pandemic H1N1	Seasonal-A	Period
Hospitalization rate (Hr) (%)	7.5	8.6	6.8	4 August to 3 September
Deaths/IPD cases (D)	29/397	23/185	6/212	August 2009
CFR (%)*	0.55	1.07	0.19	
Death/IPD cases (D)	23/398	21/217	2/181	September 2009
CFR (%)*	0.43	0.83	0.08	
Death/IPD cases (D)	13/198	13/167	0/31	October 2009
CFR (%)*	0.49	0.67	0	
Total deaths/total IPD cases (D)	65/993	57/569	8/424	1 August to 31 October 2009
Overall CFR (%)* Hr * D (%)	0.49	0.86	0.13	

* CFR  =  Hr*D.

## Discussion

As observed in some countries of southern hemisphere [14], within a short period of about 2 weeks after establishment of indigenous transmission, pandemic H1N1 became one of the predominant sub-type in Pune and it continued to co-circulate with seasonal influenza viruses. Unlike in New York City, where 90% of the samples tested were positive for pandemic H1N1 virus [15], both seasonal-A and pandemic H1N1 were represented in almost equal proportion in this study. As per our estimation there were about 11 OPD cases of pandemic H1N1 in the community for each hospitalized case. This was also considered reasonable by Wilson and Baker [10]. Unlike in Singapore [16], there was no evidence of replacement of existing seasonal circulating strains by pandemic H1N1 virus. Both seasonal and pandemic viruses continue to circulate even beyond study period (data not shown).

Clearly, both seasonal-A and pandemic H1N1 viruses were highly active and present in almost equal proportions during the study period. It provided unique opportunity to study impact of these viruses independently and also to compare their severity. The epidemic started in monsoon season and continued with cyclic pattern. This pattern is similar to that observed for seasonal influenza in tropics [17]–[23].

We believe that underreporting during initial phase of the epidemic was minimal due to the highly vigilant health department which sprang into action after the first death in Pune. Significant underreporting of actual OPD cases after the first few weeks of the infection occurred due to change in policy but IPD admissions and fatality reporting continued properly even in the middle of the epidemic. This fact was obvious as two distinct waves were seen in IPD cases but second wave was diminished in OPD data. Hospitalization ratio reported here is in line with the reports from USA and Canada [12]. The cumulative incidence of hospitalization due to pandemic H1N1 was comparable to that reported for Argentina, Australia, New Zealand etc [13].

The relationship between hospitalization and death cases observed by us and in USA and Canada [11] appears to be an ideal tool to assess the total number of case in the community. However, this will be true only if available data on hospitalization is of good quality and the conditions for significant bias are not present. The higher risk of hospitalization observed by us is significant and needs to be evaluated in different places.

The significantly higher CFR for pandemic H1N1 in comparison to seasonal-A noted in this study indicates that pandemic H1N1 is significantly more severe than seasonal-A in tropical settings like India. The rate is much higher than that reported from Canada, USA and European countries but matches with the initial CFR observed in Mexico [12]. On the other hand severity of seasonal influenza is almost similar to that reported for developed countries. Although the CFR of pandemic H1N1 is lower than that reported for ‘Spanish flu’, it is much higher than those reported for other pandemics of 20^th^ century [24]. The finding is in contrast to the reports of similar death rates between pandemic H1N1 and seasonal–A [3], [5]. Normalized CFR for each age group shows a marked increase in fatality risk with age except in age group >60 years represented by only 1 fatal case. The same pattern was also observed in Thailand [25]. Relatively few deaths in seasonal influenza group were reported but comparatively high fatality was reported in elderly persons.

The CDC proposed an important and new concept ‘Pandemic severity index’ (PSI) as a new pandemic influenza planning tool for planning at different places [26]. The PSI is based on case-fatality ratio as a single criterion to assess severity of pandemic in initial stages. Five levels of PSI, 1 to 5 were proposed to describe low to high severity. According to this classification, the pandemics of 1957 and 1968 both fit in category 2, whereas the pandemic of 1918-19 qualified as a category 5. The Pune epidemic of pandemic H1N1 falls in Category 3 of the severity index.

CFR at country level is often derived using many generalized assumptions, the validity of which is rarely proved. In a tropical country like India, large variations have been noticed in the morbidity and mortality during this pandemic. Higher incidence of influenza is associated with rainy and winter seasons though activity continues throughout the year. Therefore, different areas may experience different levels of influenza related hospitalizations and fatalities at different times. It is imperative that systematic studies be conducted in multiple places to arrive at a reasonable estimate of severity of the pandemic and seasonal influenza viruses. Mapping of the risk factors to delineate risk zones may provide better country plans.

This report on severity of influenza infection is based on actual systematically collected epidemiologic data. Such information could not be provided earlier due to the lack of dedicated surveillance of this magnitude. Earlier projections were based on estimates derived from parallel data collected from other parts of the world. The similarity of CFR between India, Mexico [2] and Thailand [25] suggests that the epidemiology of the virus in tropical settings may be different than that of temperate regions [10]. In animal experiments with ferrets also, the pandemic H1N1 virus has been found more pathogenic than seasonal influenza viruses but less pathogenic than 1918 flu virus [27]–[29].

In summary, in India, pandemic H1N1 virus was associated, with more severe disease outcomes both in terms of hospitalization and mortality. The severity of pandemic H1N1 is lower than that reported for ‘Spanish flu, 1918’ but much higher than reported for other pandemics of 20^th^ century. Comparatively, the seasonal influenza produces milder disease with much less mortality.

## Materials and Methods

### Study area

Pune, located at 18° 31′ North latitude and 73° 51′ East longitude, is one of the 35 districts in Maharashtra state, India. The population of the district is about 7.2 million and of this about 4 million live in Pune Urban Agglomeration (urban). In the present study, data on hospitalization and samples tested in laboratory were obtained only for Pune (urban). However, the data on death cases were available for Pune (urban) and also the other areas in the district, referred as Pune (rural).

### Case definitions

A suspected case of influenza like infection (ILI) was a person with acute respiratory illness who had fever or a recent history of fever and sore throat. A suspected case of pandemic H1N1 was a suspected case of ILI who also had an epidemiological link with a confirmed pandemic H1N1 virus infection. A confirmed case of pandemic H1N1 was a ILI case with laboratory confirmation of pandemic H1N1 virus by real time reverse transcriptase polymerase chain reaction (rRT-PCR). A confirmed case of seasonal-A influenza was a suspected case of ILI with laboratory confirmation of influenza-A by rRT-PCR but negative for pandemic H1N1. It included un-typable as well as seasonal H1N1 or H3N2 viruses. A confirmed case of influenza-A included the cases positive for pandemic H1N1 and seasonal -A.

### Laboratory diagnosis

Throat and nasal swabs from the suspected cases, presenting at outpatient departments (OPD) or admitted in-patient departments (IPD) of the respiratory units in city hospitals, both in public and private sectors were collected and transported to the laboratory in transport medium on ice. All samples were tested by rRT-PCR following the Centers for Disease Control and Prevention (CDC) protocol [30] for confirmation of pandemic H1N1 virus and the WHO protocol [31] for identification of seasonal influenza on ABI 7500 real time PCR machine. Diagnosis was invariably provided within 24 hours of the receipt of the samples. All patient related data including demographic details were obtained and entered in a database created for the purpose. Due to heavy work load, only limited numbers of samples, randomized, were tested for influenza B viruses. Limited samples were sub typed to understand proportional representation between seasonal H1N1 and H3N2 circulating viruses.

### Surveillance and data collection

Following the first death on 3 August, an unprecedented level of surveillance was mounted by the government to monitor influenza cases. Regular daily meetings involving senior government officials, health officials and hospital representatives were held by the district administrative authorities. Duly filled forms submitted by each hospital were reviewed and systematic reporting of number of patients screened, admitted and outcome for each hospitalized patient was ensured.

Initially, due to strong media hype and high level of government attention, the turnout of patients with any symptom of influenza was very large and almost all the affected persons presented themselves to the screening centers. Many such centers were opened across the city to facilitate active surveillance of the pandemic. Soon it was impossible to test all the cases and provide reports in a timely manner. This demanded changes in policy and the suspected patients were provided with Oseltamivir without waiting for laboratory results from the second week of September onwards. Thereafter, only limited samples, collected at the discretion of the clinicians, from OPD were submitted for testing. However, systematic sample collection from all the admitted patients was continued throughout the study period

### Calculation of case fatality ratio (CFR)

CFR, the percentage of deaths out of the total confirmed cases of the disease [25], was determined following the method suggested by Garske et al [12]. The first stage involved a short period in the beginning of the outbreak, from 4 August to 3 September, for which reliable data was available on all the cases. This data was used for determination of ratio between the hospitalized cases and the total cases, termed as hospitalization ratio. It was assumed that this hospitalization ratio will hold good for entire season of the transmission. In the second stage, only hospitalized cases and deaths were used to estimate the CFR among hospitalized cases. The CFR was estimated by multiplication of the hospitalization ratio calculated in the first stage and the CFR among hospitalized cases in later stages. Overall, CFR was computed by multiplication of the hospitalization ratio of the first stage and the CFR among hospitalized cases from 1 August to 31 September 2009. Similarly, CFR was also computed for individual months during the study.

The normalized age-specific CFR was calculated following de Silva et.al [24]. It was calculated by dividing the age distribution of all deceased patients against the age distribution of all confirmed cases and further dividing each value by the overall CFR for the total population.

On occasions, when all the samples could not be presented to the laboratory or all the presented samples could not be tested, the number of positive cases was derived by multiplying the recorded cases with the ratio obtained in the samples tested in laboratory.

The ethical clearance for the study was not required since samples were referred to us for diagnosis as a public health response to mitigate the pandemic. For the data presented in this study, the participating patient information remained anonymous.
